# Exploring the impact of the COVID-19 pandemic and UK lockdown on individuals with experience of eating disorders

**DOI:** 10.1186/s40337-020-00319-y

**Published:** 2020-08-31

**Authors:** Dawn Branley-Bell, Catherine V. Talbot

**Affiliations:** Department of Psychology, Northumbria University, Newcastle upon Tyne, England, UK

**Keywords:** Pandemics, Coronavirus, COVID-19, Eating disorders, anorexia nervosa, Bulimia nervosa, Vulnerable populations, Healthcare

## Abstract

**Background:**

The coronavirus disease 2019 (COVID-19) pandemic may raise unique challenges for individuals with experience of eating disorders. Many factors have potential for detrimental impacts on psychological wellbeing and eating disorder recovery, including: Disruption to living situations, ‘social distancing’ restrictions, difficult access to healthcare, and societal changes to food behaviours and technology usage. To date, little is known on the impact of the pandemic on this population, particularly within the UK.

**Method:**

A mixed-methods online survey was developed for the purpose of this study. Data was collected from 129 individuals currently experiencing, or in recovery from, an eating disorder during the early stages of the UK pandemic lockdown. Participants were aged between 16 and 65 years, with 121 participants identifying as female, 7 male and 1 participant preferring not to disclose their gender.

**Results:**

Findings suggest that the pandemic is having a profound, negative impact upon individuals with experience of eating disorders. Eight key themes were generated: Disruption to living situation, increased social isolation and reduced access to usual support networks, changes to physical activity rates, reduced access to healthcare services, disruption to routine and perceived control, changes to relationship with food, increased exposure to triggering messages, and positive outcomes. The results suggest detrimental impacts on psychological wellbeing including decreased feelings of control, increased feelings of social isolation, increased rumination about disordered eating, and low feelings of social support.

**Conclusions:**

Individuals with eating disorders are at significant risk of negative impacts of the pandemic. There is a vital need for interventions to support this population. Inequalities in healthcare provision were identified, emphasising a need for a more cohesive approach to remote treatment across UK healthcare services. Positive aspects of technology use were identified but the results suggest a need to address and/or limit the potential for negative impacts of public messages around food and exercise behaviours, and to co-design technologies with end-users to facilitate effective treatment.

## Plain English summary

This research uses an online survey to gather data from individuals in the UK with experience of eating disorders during the coronavirus disease 2019 (COVID-19) pandemic. The results suggest that disruptions to daily life as a result of lockdown and ‘social distancing’ may have a negative impact on individuals’ wellbeing. Negative effects may be due to changes to individuals': regular routine, living situation, time spent with friends and family, access to treatment, engagement in physical activity, relationship with food and use of technology. The research discusses how these issues can be addressed via further developments within healthcare, research, governance and policy. This could benefit those experiencing eating disorders and also mental health issues more broadly.

## Introduction

Individuals with, or in recovery from, eating disorders (EDs) are likely to be negatively impacted by the COVID-19 pandemic. ED symptoms may be exacerbated by media exposure, disruption to daily activities, social isolation, modified physical activity and sleep, and negative affect and fear of contagion [1]. The pandemic may also lower protective capacity and access to care, by restricting access to social support, treatment, and adaptive coping strategies [1]. Psychological factors linked to ED symptoms will likely be exacerbated by the pandemic and associated lockdown. For example, stress [2, 3], health anxieties and contamination fears [2, 4, 5], perceived lack of control and problems with emotional regulation [5, 6], reliance upon routine, structure [4, 5], and social isolation [3, 5].

Technology may play an important role at this pivotal time when physical socialisation is limited and virtual interaction comes into its own. Internet use has been linked to both negative and positive consequences for this population [7]. Thus, it is important to investigate the role that technology is playing at the current time. As a result of the pandemic, healthcare organisations also find themselves suddenly relying more heavily on virtual methods to deliver treatment and support. Research is already underway to identify how therapy for EDs can be translated to online delivery [8, 9]. Despite these efforts, healthcare organisations - and those receiving treatment – may face significant challenges with the move to remote healthcare [4]. For a population that can require regular in-person appointments [5], this reduction in service provision could be catastrophic.

Researchers from across the globe have started to investigate the impact of the pandemic upon individuals experiencing EDs. Early studies from Singapore and Barcelona highlight how increased anxiety is having a negative impact [2, 10]. In Australia, Phillipou et al. [11] found evidence of increased restricting, binge eating, purging, and exercise among this population. Many reports focus upon countries with greater experience of infectious disease outbreaks (e.g., [4, 5, 12]). These countries are generally more prepared for dealing with the pandemic than others [5] due to previous experience of contact tracing and disease surveillance, provision of emergency healthcare resources needed for isolation, and co-ordinated public health messaging. There are other global studies in progress, e.g., in Australia [11], the United States [13] and the Netherlands [13]. However, research within the United Kingdom (UK) is limited. Fernández-Aranda et al. [10] conducted a small, qualitative UK study (*N* = 8) describing some of the challenges faced by those with EDs during COVID-19. However, no quantitative data was reported, and the sample was specific to those with Anorexia. To the best of our knowledge, there has been no empirical work conducted with people with EDs in the UK during the pandemic. The current research addresses this, with the aim of investigating the impact of the pandemic and UK lockdown on the lives of people with experience of EDs. In addition to raising awareness, the results could influence future health service provisions, guidance and policies.

## Methods

### Sample

One hundred fifty-three participants were recruited via social media (Twitter and Facebook) using opportunistic and snowball sampling. Inclusion criteria was: Over 16 years of age, current UK resident, experience of an ED (including those in recovery). Recruitment was limited to the UK to allow the researchers to accurately identify the stage of the pandemic and the lockdown, and to ensure that data was comparable in this regard. Twenty-four participants were excluded due to not proceeding past the first page of the survey or not meeting the inclusion criteria. The final sample consisted of 129 participants, aged between 16 and 65 years (*M* = 29.27 yrs., *SD* = 8.99 yrs). One hundred twenty-one participants identified as female (93.8%), 7 male (5.4%) and 1 (0.8%) participant preferring not to disclose their gender. The majority (*N* = 80, 62%) of the sample described themselves as currently experiencing an ED (including those in a period of relapse), 8 (6.2%) described themselves as “in recovery <3 months”, 8 (6.2%) “in recovery between 3-12 months”, and 33 (25.6%) “in recovery >12 months”.

### Procedure and measures

Recruitment took place in early April 2020, 2 weeks after UK lockdown restrictions were first enforced. Participants completed an anonymous online survey containing questions around their experience of EDs and the pandemic. This included closed and open-ended questions about: the social impact of the lockdown; internet usage; food and exercise behaviours; and the overall impact of the pandemic on their EDs. Established scales were used to measure: Mental wellbeing, perceived stress, social support, perceived control and rumination (see Supplementary Material 1).

#### Short Warwick-Edinburgh mental wellbeing scale

The 7-item Short Warwick-Edinburgh Mental Wellbeing Scale (SWEMWBS: [14, 15]) was used to measure emotional and functioning aspects of mental wellbeing. Scores range from 7 to 35, with higher scores indicative of greater mental wellbeing. Cronbach’s alpha for the scale in the current study = .81.

#### Perceived stress scale

The Perceived Stress Scale (PSS: [16]) is one of the most widely applied measures of perceived stress, i.e., the degree to which an individual perceives situations within their life as stressful. The shortened 4-item version was used in the current study as it has been shown to maintain good validity and reliability [17]. The PSS-4 gives a score out of 16, with higher scores indicating greater perceived stress. Cronbach’s alpha = .72.

#### ENRICHD social support instrument

The ENRIHD Social Support Instrument (ESSI: [22, 23]) is a 7-item measure of social support, i.e., the existence or availability of people on who an individual can rely. Scores range between 8 to 34, with higher scores indicating greater social support. Cronbach’s alpha = .86.

#### Shapiro control inventory

To measure perceived control we used the Shapiro Control Inventory (SCI, [24]) general domain sense of control scale. This 16-item scale encompasses positive and negative sense of control. Scores range between 16 and 112 with higher scores indicating greater perceptions of control. Cronbach’s alpha = .86.

#### Rumination response scale for ED

The Rumination Response Scale for Eating Disorders (RRS-ED, [25]) is a 9-item measure of preoccupation with ruminative themes of eating, weight and shape. It is the first self-report measure to specifically capture ED rumination. The measure gives a total score between 9 and 36, with higher scores indicative of greater rumination. Cronbach’s alpha = .86.

### Analytical approach

A mixed methods approach was applied. Descriptive statistics, and between group comparisons (current ED group vs. recovery group) were complemented with thematic analysis of the qualitative data (using NVivo12). The second author coded the dataset independently using both an inductive and deductive approach, whereby some categories were developed prior to the analysis and others were generated from the data. Codes were collated to identify initial themes. To increase rigor, a team approach was taken with the first author providing critical feedback on themes and coding. Amendments were made where appropriate. The finalised themes were then generated and agreed upon by both authors.

## Results

### Quantitative measures

Mean scores for the five scales are provided for participants categorised into two groups: those currently experiencing an ED, and those who report being in recovery (Table 1).

**Table 1:** Participant scores for mental wellbeing, perceived stress, social support, perceived control and ED rumination

	Current ED			In Recovery		
	*N*	*M*	*SD*	*N*	*M*	*SD*
Mental Wellbeing (WEMWBS)	80	16.35*	3.24	49	17.66*	2.48
Perceived Stress (PSS)	80	14.66**	2.61	49	13.10**	2.71
Social Support (ESSI)	80	19.76*	5.71	49	21.94*	6.47
Control (SCI-general)	77	54.52**	13.15	48	62.71**	12.74
Rumination (RRS-ED)	76	22.67†	6.96	47	20.04†	7.59

Significant differences between the groups are indicated. ** *p* = .001 * *p* < .05 †*p =* borderline (.052)

Significant differences were found between the two groups for all measures except the rumination scale which just failed to reach significance (*p* = .052). The group differences are in the direction that would be expected with the recovery group showing higher mental wellbeing, lower perceived stress [2, 3], higher social support [3, 5] and higher perceived control [5, 6]. The borderline significant result was also in the expected direction with those in recovery scoring lower for ED rumination [26].

### Overall impact of the pandemic

When asked about the overall impact of the pandemic on their ED symptoms, an overwhelming majority (86.7%) reported that their symptoms had worsened as a result of the pandemic, with over 30% reporting that their symptoms were *much* worse. In contrast, only 2 participants reported a slight improvement in their symptoms (Table 2).

**Table 2:** Participants' reported overall impact of the pandemic on their ED symptoms (*N* = 129)

	*N*	%
Symptoms much worse	38	30.9
Moderately worse	48	37.2
Slightly worse	24	18.6
No change	11	8.5
Slightly better	2	1.6
Moderately better	0	0
Symptoms much better	0	0
Missing	6	4.7

We explore potential factors for this detrimental impact further in the following section.

### Themes

The analysis generated eight themes relating specifically to the effects of the pandemic and associated lockdown: (1) Disruption to living situation; (2) Increased social isolation and reduced access to usual support networks; (3) Changes to physical activity rates; (4) Reduced access to healthcare services; (5) Disruption to routine and perceived control; (6) Increased exposure to triggering messages; (7) Changes to the individual’s relationship with food; (8) Positive outcomes.

#### Disruption to living situation

Over a fifth of participants reported a change to their normal living situation due to the pandemic (Table 3). When asked whether they felt this change had affected their ED symptoms, 85.2% reported that the change had worsened their symptoms. Reasons included: Increased interpersonal stresses, hiding their ED from others, increased scrutiny and/or pressure from others to eat more, and a loss of control over diet. For example, one participant describes how their relationship is impacting upon their ED:

My relationship with my partner significantly impacts my eating behaviours and this has been worse since the pandemic*.*

Whilst hiding their ED from others left some participants reporting feelings of being “trapped”, “incredibly low” and “unhappy”:

I’m trapped at home with people who don’t know I have anorexia. I am hiding and lying constantly*.*

When describing the challenges associated with being in lockdown with partners or family members, participants tended to refer to mealtimes. Specifically, participants felt that the people they lived with were often critical of their eating behaviours:

I have much less choice of what I eat and I have to eat most of my meals in front (and in scrutiny) of others and that is causing enormous stress*.*

This pressure to eat appears to be worsened by others worrying that the individual will be unable to recover if they contract COVID-19, due to not correctly fuelling their body:

My mother is particularly concerned about me because I “do not give my body enough energy to overcome the virus” if I contracted it, and she wants me to eat more because of this. This is weighing on my mind because I absolutely do not want to do this*.*

Suddenly living with others resulted in many participants losing control over the meals they were able to eat, for example due to others taking responsibility for cooking:

My partner is making more of our meals so I’m less certain about the ingredients and amounts, so less able to track this using MyFitnessPal. I’m also less in control of what I eat for breakfast and lunch, when usually I would have full control over this in the office*.*

In the likely event of the pandemic continuing for some time, the prolonged stress of living in challenging environments could be particularly detrimental for those experiencing EDs.

#### Social isolation and reduced access to usual social support networks

The vast majority (86.4%) of our sample reported greater feelings of social isolation as a result of the pandemic (Table 3). For many individuals, spending time with friends and family represents a vital factor in their ED recovery. Lockdown has resulted in many participants being unable to access and/or physically engage with these support networks. Impacts of this include worsened ED symptoms and reduced motivation for recovery:

Unable to see friends, who are supportive and boost my mood (which affects my eating)*.*

The social aspect of eating can be particularly important for helping some individuals manage their symptoms. Going ‘out’ for food can provide an important coping mechanism to encourage them to eat. The lockdown clearly impedes this:

The MAJORITY of my “safe” eating takes place outside of the house. E.g. getting coffees, going to Pret for food I am familiar and comfortable with. There is something less shameful and “holding” for me about eating out, and that isn't the case at home. Now I have to eat more at home, on my own, and the isolation around eating and exercise is not a good place for me to be*.*

Unfortunately, isolation can also impact recovery through reduced feelings of accountability. Whilst individuals who find themselves suddenly living with others may experience increased feelings of scrutiny and pressure to recover (refer to Disruption to living situation section), the opposite can occur for those who find themselves isolated from others. Over 20% of our sample reported feeling less social pressure to recover due to the pandemic:

Not seeing those close to me who would recognise my losing weight and deterioration has put me under less pressure to challenge my ED as I can unwitnessed lose weight without challenge from others which is less pressure for me*.*

Some participants felt that they were becoming more restrictive with their diet and expressed concerns around the impacts that this could have, not only now, but to their future recovery:

I am concerned that I am becoming more rigid about what I eat when I had moved on from this. I worry it will be harder to go back to social eating*.*

Here, we are provided with a crucial reminder that the impacts of the pandemic are likely to be felt for a prolonged period of time; continuing after cessation of the lockdown and the return to ‘normality’. It is also important to recognise that ED behaviour that occurred during the pandemic may have been reinforced by repetition during a lengthy lockdown period. These reinforced or ‘habitual’ behaviours can be resistant to change.

#### Changes to physical activity rates

Participant responses to the impact of the pandemic upon physical activity were varied. The researchers initially assumed that physical activity rates may drop as a result of lockdown, and indeed almost half of our sample reported a reduction in physical activity. However, 36.5% reported an *increase* in physical activity (Table 3). Qualitative responses indicate that activity increases were primarily driven by anxieties about weight gain and a desire to counteract the effects of inactivity during lockdown:

I was doing lots before but even more now partly because I actually like it, partly to fill the time, partly from an unhealthy driven mentality. Rest days now feel unnecessary because I'm resting every day if I'm not working*.*

Not being able to freely engage in ED behaviours such as bingeing and purging provided another driver:

Now people are home with me my binge purge has reduced to every other day. However, my exercise has increased to compensate for this*.*

It can be easier for others to monitor or ‘police’ eating behaviours than it is to identify when an individual has crossed the line between healthy exercise and excessive, detrimental activity levels. It can also be easier for individuals to defend and rationalise physical activity, especially during lockdown when daily exercise was so widely discussed in government daily COVID-19 updates. Participants were aware of this tension between healthy activity and over-exercise, and reported finding it difficult to manage their relationship with exercise during lockdown:

There is part of me that wants to do all of the exercise, to ‘get thin’ and to listen to the disorder. But there is also part of me that knows I need to moderate my exercise because it’s so triggering for me*.*

For those who did report a *decrease* in activity, the same anxieties around weight gain provided a driver for engaging in restrictive eating behaviours:

Can't exercise as much / the way I was, and not getting as many steps, so being more restrictive with foods eaten, calories, and timing of eating (doing intermittent fasting again)*.*

This demonstrates that the pandemic can increase symptoms both in relation to eating behaviours and/or excessive exercise, depending upon the individual and the context they find themselves in during lockdown (Table 3).

**Table 3:** Reported changes due to COVID-19 pandemic and effect on ED symptoms

Factor	Changed due to pandemic	*N*	%	Effect of change on symptoms (of those who indicated change)	*N*	%
Living situation	Yes	27	20.9	Symptoms much worse	1	3.7
	No	98	76	Moderately worse	11	40.7
	Other	4	3.1	Slightly worse	11	40.7
	Total	129	–	No change	2	7.4
				Slightly better	1	3.7
				Moderately better	1	3.7
				Symptoms Much better	0	0
				Missing	0	0
				Total	27	–
Social isolation	Much more isolated	56	43.4	Symptoms much worse	33	28.2
	Moderately more isolated	35	27.1	Moderately worse	45	38.5
	Slightly more isolated	17	13.2	Slightly worse	24	20.5
	No change	8	6.2	No change	12	10.3
	Slightly less isolated	5	3.9	Slightly better	2	1.7
	Moderately less isolated	4	3.1	Moderately better	0	0
	Much less isolated	0	0	Symptoms Much better	0	0
	Missing	4	3.1	Missing	1	0.9
	Total	129	–	Total	117	–
Usual support network(s)	Yes	93	72.1	Symptoms much worse	14	15.1
	No	31	24	Moderately worse	26	28
	Missing	5	3.9	Slightly worse	27	29
	Total	129	–	No change	23	24.7
				Slightly better	3	3.2
				Moderately better	0	0
				Symptoms Much better	0	0
				Missing	0	0
				Total	93	–
Physical activity	Much less physical activity	36	27.9	Symptoms much worse	41	36.9
	Moderately less	15	11.6	Moderately worse	32	28.8
	Slightly less	13	10.1	Slightly worse	28	25.2
	No change	13	10.1	No change	8	7.2
	Slightly more	16	12.4	Slightly better	1	0.9
	Moderately more	13	10.1	Moderately better	0	0
	Much more	18	14	Much better	1	0.9
	Missing	5	3.9	Missing	0	0
	Total	129	–	Total	111	–

#### Reduced access to healthcare services

One of the major challenges faced by participants was a reduction in healthcare service provision and/or discrepancies in access to healthcare services. Participants reported being prematurely discharged from inpatient units, having treatment suspended or remaining on a waiting list for treatment, and receiving limited post-diagnostic support:

The pandemic meant that I was discharged suddenly and prematurely from inpatient treatment, and has also meant that my post-discharge support is limited*.*

It was clearly a distressing time for participants, and a reduction in service provision caused some participants to feel like a “*burden*”, an “*inconvenience*”, and “*forgotten*” by the government and NHS. The consequences of not being able to access professional treatment during the pandemic could be severe, and in some cases even fatal. These concerns were echoed by one participant:

People are going to die during this from their ED because they are unable to be physically monitored*.*

Unfortunately, this reduced physical monitoring links back to the aforementioned lack of accountability (refer to Social isolation and reduced access to usual social support networks section) with participants reporting feeling greater ‘opportunity’ to engage in undetected ED behaviours:

Because I no longer can go to the day service, my anorexia makes me believe it’s a chance to ‘get away’ with me e.g. losing a ton of weight*.*

Despite the reduction in service provision, some participants stated that they were still able to access professional healthcare services via technology, such as video-calling software. However, whilst online support was described as a positive factor, participants described this as falling short of treatment and support received in-person. They did not view online treatment as a direct replacement for face-to-face support, but rather the ‘next best thing’ or the ‘only alternative’ when traditional support mechanisms were unavailable:

I wouldn't usually access phone support or video chat. I dislike doing these, but felt I had to having lost my face to face support*.*

Unfortunately, participants reported discrepancies in access to services. Not all services are using technology to support individuals during the pandemic – or they have been delayed in their provision of such services. One participant described the inequality in service provision across the UK as a “*postcode lottery*”:

There is a postcode lottery in terms of what services are now providing, some patients in some areas were offered video chat straight away, my service still haven't got this up and running*.*

Remote services also raised their own, perhaps unexpected, issues. For example, some participants felt that video calling was having a detrimental effect on their ED. Seeing their own video during the call, made some participants more aware and more critical of their appearance:

Have seen myself more and made me question if I good enough or look good. Also, things have changed to online video conferencing. It’s just the amount of time seeing yourself*.*

It is vital that researchers and service providers accurately assess the appropriateness of technologies for remote service provision.

Some participants were also concerned about being asked to self-monitor by healthcare professionals, whilst relying on remote treatment. Many individuals experiencing EDs choose not to keep weighing scales in their homes as this can lead to an unhealthy fixation on their weight. This is in direct conflict with being asked to self-monitor to enable remote treatment:

Many of us trying to recover have gotten rid of our scales as for some they fuel the compulsive nature of the ED - I wonder how many will worsen due to being told to buy scales from ED services*.*

The challenges of physical monitoring - the ED service that I am under would prefer for me to have own scales and self-report weight whereas GP sees this as a big risk in itself (I would become dangerously fixated on the number)*.*

Our findings highlight discrepancies in service provision, a lack of consistency in advice between services, concerns around the suitability of some remote treatment methods, and the need to develop clear evidence-based guidelines for best practice.

#### Disruption to routine and perceived control

For many, keeping to a regular routine is vital for ED recovery and relapse prevention. Unfortunately, many routines have been significantly impacted as a result of lockdown:

Feeling frustrated because I’ve been learning how to control the bulimic symptoms and normally able to manage them. Yet the change in routine (or lack of) and general stress/anxiety is unsettling it and noticed my thoughts and behaviours changing*.*

The majority of participants (65.9%) reported some change to their usual coping mechanisms as a result of the pandemic. A lack of routine and/or distractions created more time for rumination about weight, exercise habits, and meals. Routine can also contribute to an individual’s perceived sense of control; another significant factor in recovery and general psychological wellbeing. When perceived control is low (such as during the pandemic), disordered eating can represent one thing that the individual *can* control:

Normally when I am in control over everything I am able to do better. I currently have less control of being able to go out, my studying, work etc and I feel subconsciously I am controlling this with food*.*

Due to the pandemic’s overwhelming impact on routines, we may see an increase in ED behaviours as a coping mechanism.

#### Increased exposure to triggering messages

Unsurprisingly, given the current lockdown, the majority (81.4%) of participants reported spending more time online. This is a trend seen in the population more generally, with digital spaces switching from an amenity to a necessity . When asked whether this had an effect upon their ED symptoms, over half (55%) of our sample reported that this increase in time online had worsened their symptoms (Table 4).

**Table 4:** Changes to time spent online due to the COVID-19 pandemic

Time spent online	***N***	%	Effect on ED symptoms^a^	***N***	%
Much more time	47	36.4	Symptoms much worse	11	9.9
Moderately more time	34	26.4	Moderately worse	24	21.6
Slightly more time	24	18.6	Slightly worse	27	24.3
No change	13	10.1	No change	44	39.6
Slightly less time	3	2.3	Slightly better	4	3.6
Moderately less time	3	2.3	Moderately better	0	0
Much less time	0	0	Much better	0	0
Missing	5	3.9	Missing	1	0.9
Total	129	–	Total	111	–

^a^excludes those participants who reported ‘no change’ for time spent online

There are numerous reasons why time online could have a negative impact for those experiencing an ED. For example, it is possible that without their usual social support networks and professional healthcare services, individuals may find support from less helpful sources, encounter extreme content (such as pro-anorexia content) or come across misinformation online [7]. This was supported by some participants mentioning that they accessed pro-ana websites and/or searched for “thinspo” content. However, for many participants, the main contributing factors appeared to be the increase in food and exercise-related messages dominating public discourse. For example, many social media posts refer to the general public’s “*fear of gaining weight*” during lockdown. These messages can be particularly triggering for individuals living with EDs:

There is a lot going around social media such as putting on weight during COVID-19 due to not doing as much and that is really triggering for people like myself as straight away my head thinks, that can’t happen to me! I must change the way I am towards food etc*.*

There has also been an increase in posts about physical activity, for example, people sharing information on their ‘daily exercise’ or sharing content to encourage others to stay active at home (including increased availability of workout content, e.g., the free daily YouTube workouts delivered by ‘body coach’ Joe Wicks). Excessive exercise is a major factor in many EDs and participants reported finding it difficult to cope with this increased emphasis around physical activity:

There is a heavier push on ALL social platforms to ‘stay for’ and ‘stay in shape’. Any coping mechanisms I did have before have changed. I struggle with exercise mainly, and with everything being in the spotlight it’s harder to look away from it and ignore it*.*

Content promoting exercise, also caused participants to engage in social comparisons (e.g., comparing their body to others, or comparing their level of exercise):

Watching work out videos judging myself for my lack of motivation to do anything and just how disgusting my body is compared to everyone else's out there. Everyone is doing self-improvement and I'm not*.*

These messages not only dominated social media, but also mass media stories:

I think the messages we are given through the media right now are that by eating “healthy” and “keeping active” we are somehow protecting ourselves from the virus. Actually, I don't think there is sufficient, scientifically valid evidence that this is the case. I can be rational about this, but nonetheless, I find these constant reminders quite triggering*.*

Whilst public health initiatives that promote healthy eating and exercise might be beneficial for some members of the general public, it is clear from participants’ statements that a focus on exercise and food can be triggering for people with EDs. This is particularly important when screen time is likely to be increased due to lockdown.

#### Changes to relationship with food

The lockdown has resulted in a change in food behaviours across the population, for example we have seen evidence of food hoarding leading to supply shortages. Of the participants in our study, 66.7% reported that their relationship with food has changed due to the pandemic. Lockdown can result in individuals having a shortage of food in the house (or provide them with a reason to justify a lack of supplies), or excess amounts if they are in a household that has stocked up. Any changes to food behaviours have the potential to have a significant impact upon individuals with experience of EDs. We can see some of the range of impacts in our data. Some participants raised concerns about being more likely to binge during the pandemic due to increased access to food in the household:

Being at home makes me more likely to binge as there is more food in the house*.*

Participants also reported increased levels of rumination over their food intake, which they attributed to its “*scarcity*” and being unable to purchase “*safe foods*”, referring to food that is often low in calories that a person feels is safe to eat:

I feel like I am thinking more and more about food. Part of it is scarcity and the inability to eat healthy food - this has made me feel a bit trapped and like it's all I can focus on*.*

A perceived scarcity of food during the pandemic caused some participants to feel guilty for eating food. These participants said they felt “*less deserving*” of food than other people:

Restriction can become a ‘reasonable’ thing to do because you’re saving food for later or someone else*.*

For these participants, the pandemic reinforced their ED behaviours by providing them with a way of rationalising food restriction. In addition to experiencing increased feelings of guilt, many participants also expressed that their relationship with food had become more negative and purely functional as a result of the pandemic:

Totally unenjoyable; purely functional, seeing as a ‘prescription medication’, a means to an end, almost resentful of food*.*

Interestingly, some participants used the pandemic to rationalise their food restrictions for other reasons stating that they felt they were “*less likely to get sick with anything if I don’t eat”*. This is a stark contrast from the attitudes they reported from friends and family who encourage them to eat to prevent getting sick (see Disruption to living situation section).

#### Positive outcomes

It was uplifting to also see positive signs of adaptive coping mechanisms within our data, primarily in response to the use of technology to access support. Due to reduced physical access to usual support networks, some participants reported using the internet and social media (e.g., Facebook groups, WhatsApp, and YouTube) to speak to friends, access support from ED communities, and to follow others’ journeys to/of recovery. These platforms can represent an important resource during periods of isolation. Social media appears to facilitate social connection and support, thus combatting some of the challenges of the pandemic:

Looking at support networks for bulimia and talking to friends who know I’m fearing a relapse due to this pandemic*.*

Some participants identified unexpected, positive impacts of the pandemic. For example, for some individuals the restrictions on ED behaviour attributed with changes in their living situation were identified as a positive:

Binging [sic] and purging has reduced. I was doing this 5 times a day. Now people are home with me my binge purge has reduced to every other day*.*

This stands in stark contrast with many participants’ experiences, who found changes in living situation to be incredibly stressful (see Disruption to living situation section).

Others found positives in the social distancing imposed by lockdown. For example, explaining that reduced social interaction also reduced social comparisons, thereby reducing anxiety:

Fewer social interactions with others maybe mean fewer self-comparisons with others & also not experiencing same level of anxieties about being ‘at fault’ in some way

Others reflected on their choice of clothing during the lockdown period, reporting that they wore more comfortable clothing that was less triggering of their symptoms:

I have been less critical of and aware of my body and its day to day changes. This is because I have been wearing only leggings or tracksuits and yoga bras and haven't been ‘triggered’ by uncomfortable clothing -previously my Jeans would dig in after lunch and I would fall into a spiral of thinking I'd gained weight/body checking etc*.*

However, it should be noted that these individuals are not representative of the vast majority of participants, with many experiencing increased pressures and stress as a result of the pandemic. Also some positives, such as those associated with a lack of social interaction, may not represent a healthy long-term solution.

## Discussion

Our findings highlight eight key themes describing how the COVID-19 pandemic is affecting individuals with experience of EDs: (1) Disruption to living situation; (2) Increased social isolation and reduced access to usual support networks; (3) Changes to physical activity rates; (4) Reduced access to healthcare services; (5) Disruption to routine and perceived control; (6) Changes to relationship with food; (7) Increased exposure to triggering messages; and (8) Positive outcomes.

In keeping with previous research, our findings suggest that patients (and their loved ones) may be grateful of opportunities for remote treatment and support when in-person alternatives are not available [5, 10] – something also suggested in the recent COVID-19 call to action [3]. Preliminary findings suggest that health treatment delivered remotely via technology may be effective for those experiencing EDs, however it is noted that there is still limited evidence in this regard and more research is necessary [5]. It is also noted that serious consideration must be given beyond digital interventions due to potential inequalities in access to technology [3].

Alarmingly, our data shows inequalities in UK health service provision during the pandemic, this must be addressed to ensure consistency in service provision across the UK. In addition to ensuring equality of access, our findings highlight other barriers to effective remote treatment, such as how to monitor health and recovery progress without having a detrimental impact on mental wellbeing. This includes not requiring weighing scales within the individual’s household, and providing alternatives to technologies, such as video-calling software, which may inadvertently make users critical of their appearance (this supports previous findings (e.g., [8, 10]). In other instances, software may have the required functions available - such as an option to 'hide self-view' - but users may not be aware of this; increasing awareness or setting this option as a default should be considered. Clear guidelines are required for the design and implementation of appropriate remote/digital health services.

Our findings demonstrate the importance of social support from friends and family. Social relationships can have a protective buffering effect from stressful events [3]. This protective effect can be diminished during lockdown periods. Future research should look to identify ways to minimise the detrimental effects of physical distancing, and develope and/or encourage alternative positive coping mechanisms.

Media coverage and social media posts were a source of anxiety for our participants, due to a public preoccupation with food, weight gain and exercise. Although positive messages about diet and exercise can be beneficial for the majority of the population, it is important for healthcare and government to acknowledge that these can be triggering or upsetting for vulnerable populations, and work to establish guidelines to help address this. Risk-elevating messages designed to nudge individuals towards social distancing and safe behaviour can also lead to increased anxiety and reduced feelings of control over the situation; again, factors linked to negative impacts on psychological wellbeing and ED symptoms. As described by Holmes et al. [3] ‘research is needed to inform future approaches, including strategies to help individuals to stay informed by authoritative sources, prevent over-exposure to media, and mitigate and help manage the effect of viewing [ …] traumatic content’ (p. 552). Our findings suggest a need for clear guidelines - driven by the insight of those with lived experience - around media reporting, social media policies and positive strategies to manage emotional and psychological consequences.

Crucially, our findings illustrate why we must not underestimate the longevity of the impact of the pandemic. Individuals with experience of EDs will likely experience a long-term effect on their symptoms and/or recovery. It is important that this is recognised by healthcare services, and beyond, in order to offer the necessary resources to support this vulnerable population now and on an on-going basis.

The lessons learned as a consequence of the COVID-19 pandemic have the potential to be relevant to other public health emergencies, other ED populations, and to future circumstances which involve periods of lockdown, food shortages, and/or social isolation. As there are international studies in progress (e.g., [11]), cross-cultural comparisons could be an interesting area for future research – particularly in relation to how different countries have addressed ED treatment and healthcare during, and after, the pandemic.

### Limitations

While this study provides an in-depth examination of the impact of the pandemic on the lives of people with EDs, it does have some limitations. Firstly, all participants were recruited via social media, resulting in the sample being biased toward those who have access to and are familiar with technology. This opportunistic approach may also have resulted in the sample being biased towards people who are currently experiencing difficulties, rather than those who are not facing challenges related to their EDs. Secondly, information about specific diagnoses was not collected. This information could be collected in future research to enable comparisons between different groups of people experiencing EDs.

Finally, data were only collected at a single time point during the early stages of the UK lockdown. The pandemic will likely continue for some time, potentially having long-lasting impacts upon the lives of individuals experiencing EDs. Thus, longitudinal research is required to investigate the long-term effects of the pandemic on this population.

Due to not having a baseline measure (i.e., pre-pandemic) it is not possible to make assumptions about the impact of the pandemic on the quantitative scale scores within our sample. Again, longitudinal research could be beneficial in this regard - allowing the comparison of data from the start of lockdown with data from later time points. This would help identify the ongoing, and potentially evolving, impact of the pandemic as it progresses. The researchers are planning a follow-up study to address this. Future research may also wish to include further quantitative analysis of the relationships between the facilitators (e.g., low perceived control) and barriers to ED (high social support) behaviour during periods of lockdown.

## Conclusions

In summary, this research suggests that the ED population is at significant risk of negative impacts of the pandemic; the consequences of which may be felt long after a societal return to ‘normality’. There is a vital need for interventions to support this population during the current pandemic and beyond. Interventions should be co-designed with end-users to facilitate effective treatment. Our findings also highlight inequalities in healthcare provision that must be addressed; a more cohesive approach to remote treatment is required across all UK healthcare services. Positive aspects of technology use were identified but it is also necessary to consider potential negative impacts of public messages around food and exercise behaviours.

## Supplementary information

**Additional file 1:** **Supplementary Material 1.** Copy of survey items. Copy of all items included in the online survey for this study

## Data Availability

The anonymised datasets used and/or analysed during the current study are available from the corresponding author on reasonable request.

## Abbreviations

COVID-19: Coronavirus Disease 2019
ED: Eating Disorder
ESSI: ENRICHD Social Support Instrument
NHS: National Health Service
PSS: Perceived Stress Scale
RRS-ED: Rumination Response Scale for Eating Disorders
SCI: Shapiro Control Inventory
WEMWBS: Warwick-Edinburgh Mental Wellbeing Scale
