# Supplementary material for: Exploring the impact of the COVID-19 pandemic and UK lockdown on individuals with experience of eating disorders
Source: J Eat Disord. 2020 Aug 31;8:44. doi: 10.1186/s40337-020-00319-y (PMC7444862; doi:10.1186/s40337-020-00319-y)
Supplement: Supplementary file 1 — Additional file 1: Supplementary Material 1. Copy of survey items. Copy of all items included in the online survey for this study [file 40337_2020_319_MOESM1_ESM.pdf]

Supplementary Material 1. Copy of Survey Items

Exploring the impact of COVID-19 on the Lives of People with Experience of Eating Disorders

We would like to invite you to take part in a study about the impact of COVID-19 on the lives of individuals who have experience of eating disorders.
You are eligible to participate providing you are over 16 years of age, live in the UK, have home access to the internet (this can be by a smartphone, computer or tablet) and self-identify as experiencing an eating disorder or being in recovery from an eating disorder (this does not need to have been formally diagnosed by a medical professional).

What does participation involve? If you decide to take part, you will be asked to participate in an online survey lasting approximately 20 minutes. This survey is confidential and anonymous. The questions will ask you about the impact of COVID-19 on your daily life, including your social interactions and your use of technology. Some questions will ask about your eating disorder. You can choose not to answer any questions that you feel uncomfortable answering.

You will also be invited to participate in two follow-up surveys. You will receive the invitation to participate in the second survey by e-mail around 4-6 weeks after completing this first survey. You will receive an invitation to complete the final survey once the pandemic is regarded as under control with social isolation/distancing ceasing.

How is my data stored? We take confidentiality and anonymity very seriously. Only members of the research team will have access to the data. Your data will be anonymised and stored against an anonymous ID code. We will securely store the data that we collect and we will comply with the General Data Protection Regulation (GDPR) in our dealings with the data. Your anonymised data may be used for academic publications such as journal articles and conference presentations. You have the right to withdraw from the study at any point by contacting the researcher.

We ask for your e-mail address solely for the purpose of sending the invitation to participate in the follow-up surveys. Your e-mail address will not be linked to your personal data and will not be used for any other purpose.

Who has reviewed this study? This study has been approved by the Northumbria University Psychology Department Research Ethics Committee.

If you have any questions or would like further information, please contact any of the following:

<< deleted for anonymous review >>

If you would like any advice or information relating to eating disorders please contact your GP or contact Beat, the UK's leading eating disorder charity:

Beat Helpline: 0808 801 0677 Beat dedicated Studentline: 0808 801 0811 Beat dedicated Youthline: 0808 801 0711
Beat also offer one-to-one webchat and further information on their website www.beateatingdisorders.org.uk

Q1 I have carefully read and understood the Participant Information Sheet. I have had an opportunity to ask questions and discuss this study and I have received satisfactory answers. I understand I am free to withdraw from the study without having to give a reason for withdrawing. I agree to take part in this study. I understand that my participation is confidential, and I will not be identifiable in any published papers or reports. If you agree with all of the above statements, please indicate your consent by clicking the consent button below. If you do not consent to take part, you may just close this window.

○ I consent to take part (1)

End of Block: Default Question Block

Start of Block: Block 1

*

Q4 Age

____

Q5 Gender

○ Male (1)

○ Female (2)

○ Other (3)

○ Prefer not to disclose (4)

Q6 In which country do you currently reside?

▼ Afghanistan (1) ... Zimbabwe (1357)

Q7 How would you describe your current situation in relation to eating disorder symptoms?

- ○ Currently experiencing an eating disorder (or in a period of relapse from recovery) (1)
- ○ In recovery from an eating disorder (in recovery for less than 3 months) (2)
- ○ In recovery from an eating disorder (in recovery between 3-12 months) (3)
- ○ In recovery from an eating disorder (in recovery for over 1 year) (4)
- ○ I do not have an eating disorder (5)

Skip To: End of Survey If How would you describe your current situation in relation to eating disorder symptoms? = I do not have an eating disorder

Q8 Current living situation during COVID-19 pandemic

- ○ Living with friend(s) (1)
- ○ Living with partner or spouse (2)
- ○ Living with parent(s)/legal guardian(s) (4)
- ○ Living alone (5)
- ○ Other (Please explain) (7) ____

Q9 Is this your normal living situation or has this changed due to COVID-19?

- ◯ Yes, this is normal (1)
- ◯ No, this has changed due to the COVID-19 pandemic (2)
- ◯ Other (please explain) (3) ____

Skip To: End of Block If Is this your normal living situation or has this changed due to COVID-19? != No, this has changed due to the COVID-19 pandemic

Q10 How do you think **this change in your living situation** has affected your eating disorder symptoms?

- ◯ It has made my symptoms MUCH WORSE (1)
- ◯ Moderately Worse (2)
- ◯ Slightly Worse (3)
- ◯ About the same (no change) (4)
- ◯ Slightly better (5)
- ◯ Moderately better (6)
- ◯ It has made my symptoms MUCH BETTER (7)

End of Block: Block 1

Start of Block: Block 2

Q11 Please select the response that best describes how you have been feeling over the last 2 WEEKS:

	None of the time (1)	Rarely (2)	Some of the time (3)	Often (4)	All of the time (5)
I've been feeling optimisic about the future (1)	○	○	○	○	○
I've been feeling useful (2)	○	○	○	○	○
I've been feeling relaxed (3)	○	○	○	○	○
I've been dealing with problems well (4)	○	○	○	○	○
I've been thinking clearly (5)	○	○	○	○	○
I've been feeling close to other people (6)	○	○	○	○	○
I've been able to make my own mind up about things (7)	○	○	○	○	○

Q12 The below questions ask about your feelings and thoughts during the LAST MONTH. Please select the response that most accurately represents how often you have felt or thought this way:

	Never (1)	Almost never (2)	Sometimes (3)	Fairly Often (4)	Very Often (5)
How often have you felt that you were unable to control the important things in your life? (1)	○	○	○	○	○
How often have you felt confident about your ability to handle your personal problems? (2)	○	○	○	○	○
How often have you felt that things were going your way? (3)	○	○	○	○	○
How often have you felt difficulties were piling up so high you could not overcome them? (4)	○	○	○	○	○

Q13 Please read the following statements and select the response that most closely describes your current situation:

	None of the time (1)	A little of the time (2)	Some of the time (3)	Most of the time (4)	All of the time (5)
Is there someone avaliable to whom you can count on to listen to you when you need to talk? (1)	○	○	○	○	○
Is there someone avaliable to you to give you good advice about a problem? (2)	○	○	○	○	○
Is there someone avaliable to you who shows you love and affection? (3)	○	○	○	○	○
Is there someone avaliable to help with daily chores? (4)	○	○	○	○	○
Can you count on anyone to provide you with emotional support (talking over problems or helping you make a difficult decision)? (5)	○	○	○	○	○

Do you have as much contact as you would like with someone you feel close to, someone in whom you can trust and confide in? (6)	○	○	○	○	○

End of Block: Block 2

Start of Block: Block 3

Q14 Please read the following statements and select the response that most closely describes your current situation:

	Strongly disagree (1)	Disagree (2)	Somewhat disagree (3)	Neither agree nor disagree (4)	Somewhat agree (5)	Agree (6)	Strongly agree (7)
I am able to calmly accept that which I am not able to change or alter. (1)	○	○	○	○	○	○	○
I am able to act assertively and decisively to try to change or alter what I want to. (2)	○	○	○	○	○	○	○
I am aware of my own feelings and motivations and recognize how they affect me. (3)	○	○	○	○	○	○	○
I take appropriate responsibility for that over which I have control. (4)	○	○	○	○	○	○	○
I make the appropriate amount of effort and have sufficient discipline to reach my goals. (5)	○	○	○	○	○	○	○

I have the skills and ability to reach my goals. (6)	○	○	○	○	○	○	○
I lose control of myself. (7)	○	○	○	○	○	○	○
I lack control of my environment (other people, situations). (8)	○	○	○	○	○	○	○
I feel that I am losing control in areas where I once had control. (9)	○	○	○	○	○	○	○
I have a positive sense of control in my life. (10)	○	○	○	○	○	○	○
If I decide to, I have the ability to make changes in order to gain more control over my life. (11)	○	○	○	○	○	○	○
I am able to choose and make decisions about the important things in my life. (12)	○	○	○	○	○	○	○

I am able to set clear, realistic, and meaningful goals. (13)	○	○	○	○	○	○	○
I have the right degree of self-control. (14)	○	○	○	○	○	○	○
I am too passive and helpless. (15)	○	○	○	○	○	○	○
Others have too much control over me. (16)	○	○	○	○	○	○	○

End of Block: Block 3

Start of Block: Block 4

Q16 Do you feel more of less socially isolated as a result of the COVID-19 pandemic?

○ I feel MUCH LESS socially isolated (20)

○ Moderately less isolated (21)

○ Slightly less isolated (22)

○ About the same (no change) (23)

○ Slightly more isolated (24)

○ Moderately more isolated (25)

○ I feel MUCH MORE socially isolated (26)

Skip To: Q18 If Do you feel more of less socially isolated as a result of the COVID-19 pandemic? = About the same (no change)

Q17 How do you think **this change in your feelings of social isolation** has affected your eating disorder symptoms?

- It has made my symptoms MUCH WORSE (1)
- Moderately Worse (2)
- Slightly Worse (3)
- About the same (no change) (4)
- Slightly better (5)
- Moderately better (6)
- It has made my symptoms MUCH BETTER (7)

Q18 Do you find that due to the COVID-19 pandemic, there have there been any changes to your eating disorder support network? For example do you find that you are...

	No (1)	Somewhat (2)	Yes (3)
Talking to different friends, compared to usual? (9)	○	○	○
Accessing different help and advice networks (e.g., online websites, forums, groups), compared to usual? (10)	○	○	○
Interacting with different family members, compared to usual? (11)	○	○	○

Skip To: End of Block If Do you find that due to the COVID-19 pandemic, there have there been any changes to your eating d... [No] (Count) = 3

Q19 How do you think **this change in your support network** has affected your eating disorder symptoms?

- ○ It has made my symptoms MUCH WORSE (1)
- ○ Moderately Worse (2)
- ○ Slightly Worse (3)
- ○ About the same (no change) (4)
- ○ Slightly better (5)
- ○ Moderately better (6)
- ○ It has made my symptoms MUCH BETTER (7)

End of Block: Block 4

Start of Block: Block 5

Q20 How has COVID-19 affected your level of physical activity/exercise?

- ○ Much LESS physical activity (1)
- ○ Moderately less (2)
- ○ Slightly less (3)
- ○ About the same (no change) (4)
- ○ Slightly more (5)
- ○ Moderately more (6)
- ○ Much MORE physical activity (7)

Skip To: Q22 If How has COVID-19 affected your level of physical activity/exercise? = About the same (no change)

Q21 Do you think **this change in your level of physical activity** has affected your eating disorder symptoms?

- ◯ It has made my symptoms MUCH WORSE (1)
- ◯ Moderately Worse (2)
- ◯ Slightly Worse (3)
- ◯ About the same (no change) (4)
- ◯ Slightly better (5)
- ◯ Moderately better (6)
- ◯ It has made my symptoms MUCH BETTER (7)

Q22 Do you spend more of less time online as a result of the COVID-19 pandemic?

- ◯ Much LESS time online (1)
- ◯ Moderately less (2)
- ◯ Slightly less (3)
- ◯ About the same (no change) (4)
- ◯ Slightly more (5)
- ◯ Moderately more (6)
- ◯ Much MORE time online (7)

Skip To: Q24 If Do you spend more of less time online as a result of the COVID-19 pandemic? = About the same (no change)

Q23 Do you think **this change in time spent online** has affected your eating disorder symptoms?

- ○ It has made my symptoms MUCH WORSE (1)
- ○ Moderately Worse (2)
- ○ Slightly Worse (3)
- ○ About the same (no change) (4)
- ○ Slightly better (5)
- ○ Moderately better (6)
- ○ It has made my symptoms MUCH BETTER (7)

Q24 What type of activities do you find yourself doing online at the moment? Please provide as much detail as you can (and which you are comfortable providing).

____

____

____

____

____

Q25 What proportion of the time you spend online, do you find yourself talking about, reading about, accessing and/or sharing anything to do with eating disorders?

- ◯ Never (1)
- ◯ Some of the time (around 25% of the time) (2)
- ◯ About half the time (3)
- ◯ Most of the time (around 75% of the time) (4)
- ◯ Always (5)

Q26 Is this more or less than usual (i.e., your normal amount prior to the COVID-19 pandemic)?

- ◯ Much LESS time spent accessing things about eating disorders (1)
- ◯ Moderately less (2)
- ◯ Slightly less (3)
- ◯ About the same (no change) (4)
- ◯ Slightly more (5)
- ◯ Moderately more (6)
- ◯ Much MORE time accessing things about eating disorders (7)

Display This Question:

If What proportion of the time you spend online, do you find yourself talking about, reading about,... != Never

Q27 How often do you do each of the following?

	Never (1)	Sometimes (around 25% of the time) (2)	About half the time (3)	Most of the time (around 75% of the time) (4)	Always (5)
Offer online social support to others about eating disorders (1)	○	○	○	○	○
Receive online social support from others about your eating disorder (2)	○	○	○	○	○
Seek online advice and guidance about your eating disorder (3)	○	○	○	○	○
Engage in other online activities related to eating disorders (please explain) (4)	○	○	○	○	○

Q28 Please select the response that best reflects how often you do each of the following:

	Almost Never (1)	Rarely (2)	Sometimes (3)	Often (4)	Almost Always (5)
Think ‘why can't I handle my eating better?’ (1)	○	○	○	○	○
Think ‘why do I have problems with controlling my eating, weight and/or shape when other people don't?’ (2)	○	○	○	○	○
Think ‘why do I have such issues with my eating, weight and/or shape?’ (3)	○	○	○	○	○
Think ‘why do I always react this way around food?’ (4)	○	○	○	○	○
Think about a recent meal time wishing it had gone better. (5)	○	○	○	○	○
Consider your personality to understand why you have to try and control your eating, weight and/or shape. (6)	○	○	○	○	○

Go away by yourself and think about why you try to control your eating, weight and/or shape. (7)	○	○	○	○	○
Write down what you think about your eating, weight and/or shape and analyse it. (8)	○	○	○	○	○
Go someplace alone to think about your feelings concerning your eating, weight and/or shape. (9)	○	○	○	○	○

End of Block: Block 5

Start of Block: Block 6

Q29 **Overall, how do you think the COVID-19 pandemic** has affected your eating disorder symptoms?

- ◯ It has made my symptoms MUCH WORSE (1)
- ◯ Moderately worse (2)
- ◯ Slightly worse (3)
- ◯ About the same (no change) (4)
- ◯ Slightly better (5)
- ◯ Moderately better (6)
- ◯ It has made my symptoms MUCH BETTER (7)

Skip To: Q31 If Overall, how do you think the COVID-19 pandemic has affected your eating disorder symptoms? = About the same (no change)

Q30 Why do you think this is the case?

____

____

____

____

____

Q31 Has your relationship with food changed since the COVID-19 pandemic?

- ◯ Yes (please explain) (1) ____
- ◯ No (2)

Q32 Do you feel less or more social pressure to recover from your eating disorder as a result of the COVID-19 pandemic?

- ○ Much LESS social pressure to recover (1)
- ○ Moderately less (2)
- ○ Slightly Less (3)
- ○ About the same (no change) (4)
- ○ Slightly more (5)
- ○ Moderately more (6)
- ○ Much MORE social pressure to recover (7)

Q33 Do you find you are using the same coping mechanisms for your ED symptoms as you were before the COVID-19 pandemic?

- ○ Yes (1)
- ○ Somewhat (2)
- ○ No (3)

Skip To: Q35 If Do you find you are using the same coping mechanisms for your ED symptoms as you were before the... = No

---

Q34 Why do you feel there have been some changes in your coping mechanisms? Can you explain what these changes are?

____

____

____

____

____

Q35 Before you finish the survey, is there anything else you would like to share with the researchers. For example, have you experienced any other challenges relating to your eating disorder as a result of the COVID-19 pandemic?

____

____

____

____

____

End of Block: Block 6

Start of Block: Block 7

PARTICIPANT DEBRIEF SHEET Thank you for taking part in this research. The data gathered during these online surveys will provide further insight into the impact of the COVID-19 pandemic on individual well-being. We understand that as a result of the pandemic you may experience changes to your social situation, interaction with others and changes to your everyday routine. The findings of these surveys can help us to understand the positive and/or negative impacts (if any) that these changes can have for everyone, including those who have previously – or are currently – experiencing an eating disorder.

We will send you an e-mail in a few weeks to invite you to take part in a follow-up online survey.

If you have any questions concerning the study or you would like to withdraw your data from the project, please contact one of the researchers:

<< deleted for anonymous review >>

If you would like any advice or information relating to eating disorders please contact your GP or contact Beat, the UK's leading eating disorder charity:

Beat Helpline: 0808 801 0677 Beat dedicated Studentline: 0808 801 0811 Beat dedicated Youthline: 0808 801 0711
Beat also offer one-to-one webchat and further information on their website www.beateatingdisorders.org.uk

End of Block: Block 7
